# Lifestyle and Psychosocial Determinants of Type 2 Diabetes Risk Across Adulthood: The Role of Burnout in a Large Occupational Cohort

**DOI:** 10.3390/medsci14050546

**Published:** 2026-09-03

**Authors:** Aina Gabriela Valiente Pizá, Pedro Juan Tárraga López, Ángel Arturo López-González, Irene Coll Campayo, Carla Busquets-Cortés, José Ignacio Ramírez-Manent

**Affiliations:** 1 Primary Care, Balearic Health Service, 07010 Palma, Spain; 2 Faculty of Medicine, University of Castilla la Mancha, 02008 Albacete, Spain; 3 ADEMA University School, University of the Balearic Islands, 07009 Palma, Spain; i.coll@eua.edu.es (I.C.C.);

**Keywords:** burnout, type 2 diabetes, occupational health, lifestyle, Mediterranean diet, physical activity, risk prediction

## Abstract

Background: Lifestyle behaviors and psychosocial factors are key determinants of cardiometabolic health across the life course. Beyond traditional risk factors, emerging evidence suggests that chronic stress and burnout may influence metabolic risk through behavioral pathways such as physical inactivity and poor diet. However, their joint contribution to type 2 diabetes risk in working populations remains insufficiently understood. Objectives: Our objectives were to examine the joint contribution of lifestyle habits, sociodemographic factors, and burnout to type 2 diabetes risk, and to explore potential behavioral pathways linking psychosocial stress to metabolic risk in a large adult population. Methods: A cross-sectional study was conducted in 92,480 Spanish workers undergoing routine occupational health assessments between 2021 and 2023. Type 2 diabetes risk was assessed using validated tools (FINDRISC, QDScore, CANRISK). Lifestyle indicators included adherence to the Mediterranean diet and physical activity levels; burnout was assessed using the Burnout Assessment Tool (BAT). Multivariable logistic regression models were used to evaluate associations between determinants and diabetes risk. Mediation analyses were performed to assess the contribution of lifestyle behaviors to the association between burnout and diabetes risk, and predictive performance analyses evaluated the incremental value of burnout. Results: Burnout was independently associated with a higher probability of moderate-to-high diabetes risk across all screening tools. Exploratory cross-sectional mediation analyses indicated that lifestyle behaviors, particularly physical inactivity and low adherence to the Mediterranean diet, statistically accounted for approximately 45% of the observed association. Adding burnout to the models significantly improved discrimination and reclassification of moderate-to-high diabetes-risk classification. In addition, higher diabetes risk was consistently observed among individuals from lower socioeconomic groups and those with unhealthy lifestyle profiles. Conclusions: Lifestyle behaviors and psychosocial stress were associated with estimated diabetes risk across adulthood. Burnout was independently associated with a higher probability of moderate-to-high diabetes-risk classification, with exploratory analyses suggesting that lifestyle behaviors may statistically account for part of this association. These cross-sectional findings highlight the potential relevance of considering both behavioral and psychosocial factors when studying cardiometabolic risk in working populations, while prospective studies are needed to establish temporal and causal relationships.

## 1. Introduction

Type 2 diabetes mellitus (T2DM) is a complex chronic disease characterized by hyperglycemia resulting from a combination of insulin resistance and progressive β-cell dysfunction. It accounts for more than 90% of all diabetes cases worldwide and remains a major public health concern, affecting an estimated 540 million adults worldwide in 2021 [1], a figure projected to rise to 643 million by 2030 and 783 million by 2045 [2]. The pathophysiology of T2DM involves multiple interrelated processes, including adipose tissue inflammation, mitochondrial dysfunction, and chronic oxidative stress, all of which contribute to systemic insulin resistance [3,4,5,6].

Diagnosis of T2DM traditionally relies on biochemical parameters such as fasting plasma glucose (≥126 mg/dL), 2-h plasma glucose during an oral glucose tolerance test (≥200 mg/dL), or glycated hemoglobin (HbA1c ≥ 6.5%) [7,8,9]. However, the long asymptomatic phase of prediabetes and early T2DM necessitates population-based screening tools to identify individuals at high risk before overt disease develops [10]. To this end, several non-invasive risk assessment tools have been developed and validated, including the Finnish Diabetes Risk Score (FINDRISC) [11], the QDiabetes Risk Score (QDScore) [12], and the Canadian Diabetes Risk Questionnaire (CANRISK) [13]. These tools have demonstrated good predictive performance across diverse populations and are widely used to identify individuals at increased risk of developing T2DM [14,15,16,17].

FINDRISC, QDScore, and CANRISK are among the most widely validated non-invasive tools for estimating future T2DM risk. Although they differ in the variables included, all combine demographic, clinical, and lifestyle information to identify individuals at increased risk and support early preventive strategies in both community and occupational health settings [18,19,20].

The consequences of T2DM extend far beyond glucose metabolism. Chronic hyperglycemia contributes to the development of microvascular complications such as retinopathy, nephropathy, and neuropathy, as well as macrovascular diseases including myocardial infarction and stroke [21,22,23,24,25]. Moreover, T2DM imposes a substantial economic burden through increased healthcare costs, productivity loss, and disability-adjusted life years (DALYs) [26,27,28].

In recent years, growing attention has been directed toward the role of psychosocial stressors as upstream determinants of metabolic health. In particular, work-related stress has emerged as a relevant contributor to cardiometabolic disorders. Burnout syndrome, defined by the World Health Organization (WHO) as a state of emotional exhaustion, depersonalization, and reduced professional efficacy resulting from chronic unmanaged workplace stress [29], has gained recognition as a potential risk factor for T2DM and other cardiometabolic conditions [30,31].

Burnout affects a substantial proportion of the working population, with prevalence estimates reaching up to 30% in high-income countries, particularly among healthcare, education, and service sector workers [32,33,34]. Burnout can be assessed using several validated instruments, including the Burnout Assessment Tool (BAT), which evaluates exhaustion, mental distance, cognitive impairment, and emotional impairment [35,36]. From a biological perspective, burnout has been associated with increased sympathetic activity, elevated cortisol levels, and systemic inflammation, mechanisms that may contribute to insulin resistance and impaired glucose metabolism [37,38,39]. Previous studies have also reported cross-sectional associations between burnout and metabolic syndrome or related metabolic alterations [40,41].

Importantly, burnout may be linked to metabolic risk through both direct and behavioral pathways. Chronic occupational stress may contribute directly to metabolic dysregulation through sustained activation of neuroendocrine and inflammatory responses. In parallel, emotional exhaustion and reduced coping resources may be associated with less favorable health behaviors, including reduced physical activity and poorer dietary patterns [42,43,44]. These behaviors are themselves established determinants of type 2 diabetes risk and may therefore represent plausible intermediate pathways linking burnout with elevated diabetes risk. Based on this conceptual framework, physical inactivity and low adherence to the Mediterranean diet were specified a priori as potential behavioral mediators of the association between burnout and estimated type 2 diabetes risk. Given the cross-sectional design, however, these pathways were examined as hypothesized statistical mechanisms rather than established temporal or causal sequences. Occupational environments characterized by high demands, low control, and prolonged working hours may further exacerbate these behavioral and metabolic pathways [45].

In parallel, sociodemographic factors such as age, sex, educational level, and social class play a crucial role in shaping diabetes risk. Lower socioeconomic status has been consistently associated with higher prevalence of obesity, reduced access to preventive healthcare, and increased exposure to obesogenic environments [46,47]. Lifestyle behaviors—including tobacco use [48], adherence to the Mediterranean diet [49,50], and regular physical activity [51]—are well-established modifiable determinants of T2DM risk, reinforcing the importance of preventive strategies targeting behavioral factors.

Despite the growing body of evidence linking psychosocial, sociodemographic, and lifestyle factors to T2DM, few studies have comprehensively examined their joint contribution using multiple validated risk scales in large occupational populations. This evidence gap is particularly relevant in Spain, where large occupational cohorts provide an opportunity to examine how psychosocial, lifestyle, and sociodemographic factors jointly influence diabetes risk.

The present study aimed to evaluate the joint contribution of sociodemographic characteristics, lifestyle behaviors, and burnout to estimated T2DM risk in a large population of Spanish workers. In addition, based on the hypothesized conceptual pathway described above, we explored whether physical inactivity and low adherence to the Mediterranean diet statistically accounted for part of the association between burnout and estimated diabetes risk. We also assessed the incremental predictive value of burnout across three validated diabetes risk tools.

## 2. Methods

### 2.1. Study Design and Population

This cross-sectional study was conducted using data collected from Spanish workers who voluntarily attended occupational health assessments between January 2021 and December 2023. The study protocol received formal approval from the Ethics Committee of the Balearic Islands (Comité de Ética de la Investigación de las Islas Baleares, CEI-IB), under reference number IB 4383/20 (approval date: 26 November 2020). Eligible workers attending routine occupational health assessments during the study period were consecutively recruited through a national occupational health surveillance program covering a wide range of economic sectors. All individuals included in the study were aged between 18 and 69 years, actively employed, and provided informed consent for their data to be used for research purposes.

### 2.2. Inclusion and Exclusion Criteria

**Figure 1:**
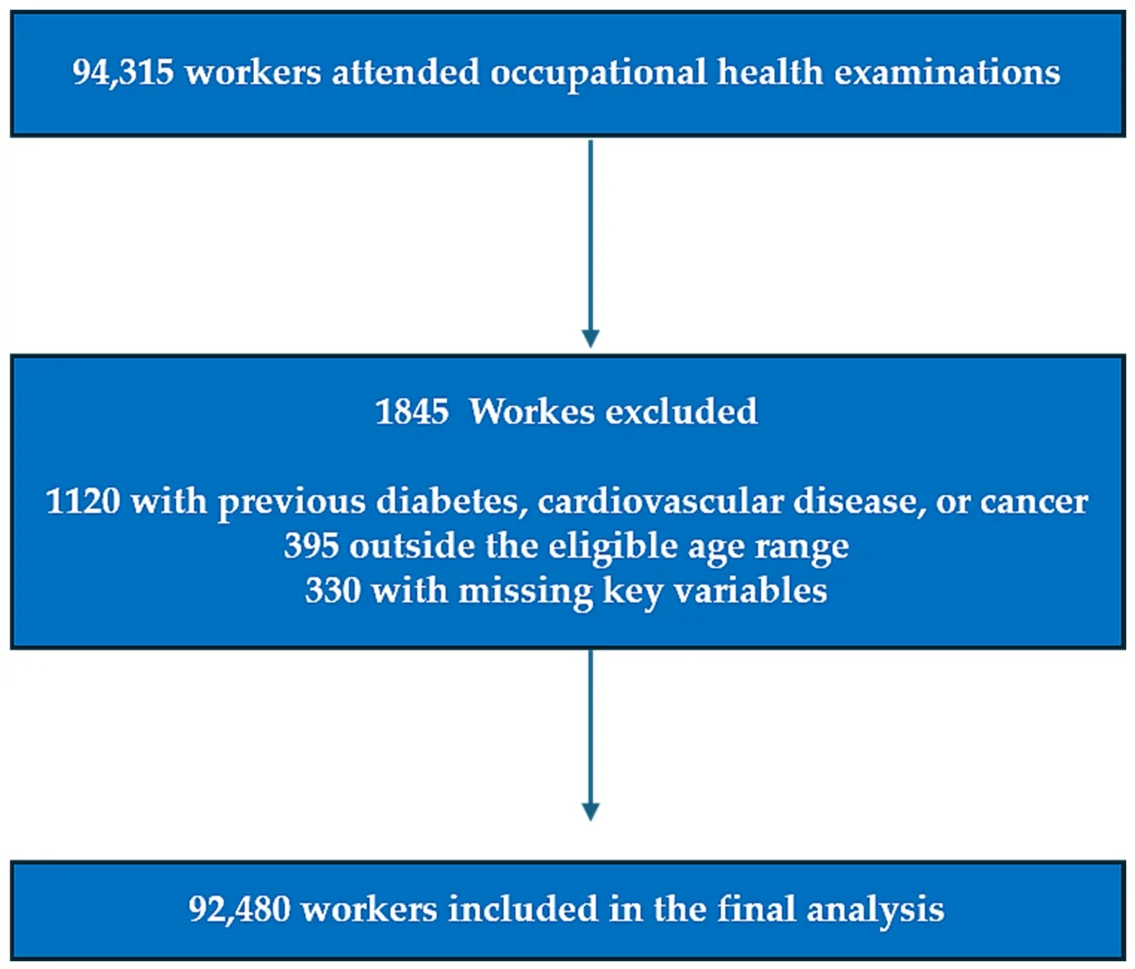
Flowchart of the study population selection process. Of the 94,315 workers initially assessed, 1845 were excluded because of previous diabetes, cardiovascular disease or cancer, age outside the eligible range, or missing key variables. The final analytical sample comprised 92,480 participants with complete data for the assessment of type 2 diabetes risk, lifestyle factors, and burnout.

Participants were eligible if they were aged 18–69 years, actively employed at the time of data collection, and had complete data for all variables required to calculate diabetes risk scores and burnout status. Participants were excluded if they were pregnant, had a previous diagnosis of type 2 diabetes mellitus, cardiovascular disease, or cancer, were receiving antidiabetic treatment, or had missing data for any key sociodemographic, clinical, or behavioral variables included in the analyses (Figure 1).

### 2.3. Clinical, Anthropometric, and Biochemical Measurements

Anthropometric, clinical, and biochemical measurements were obtained by trained occupational health personnel following standardized measurement procedures. Body weight and height were measured using a SECA 700 column scale equipped with a SECA 220 telescopic height rod (SECA, Hamburg, Germany), with participants wearing light clothing and without shoes. Waist circumference was measured using a flexible, non-stretchable SECA 200 measuring tape (SECA, Hamburg, Germany), with participants standing upright, feet together, and abdomen relaxed; the measurement was taken horizontally at the midpoint between the lowest rib and the iliac crest. Hip circumference was measured at the widest point of the buttocks, with the measuring tape maintained horizontally.

Blood pressure was measured using a calibrated OMRON M3 automatic sphygmomanometer (OMRON Healthcare, Kyoto, Japan) after participants had rested for at least 10 min in a seated position. Three measurements were obtained at 1-min intervals, and the mean of the three measurements was used in the analyses.

Venous blood samples were obtained after a 12-h overnight fast and analyzed using a SYNCHRON CX9 PRO autoanalyzer (Beckman Coulter, Brea, CA, USA). Fasting glucose, total cholesterol, and triglycerides were determined using automated enzymatic methods. HDL cholesterol was determined using a precipitation-based method, and LDL cholesterol was calculated using the Friedewald equation when triglyceride concentrations were <400 mg/dL; direct laboratory determination was used when calculation was not applicable. Laboratory analyses were performed according to standardized procedures and the internal quality-control protocols routinely implemented by the participating laboratories.

Biochemical measurements were used to characterize the study population descriptively and were not used to define glycemic status or the primary study outcomes. Although fasting glucose measurements were available, HbA1c and oral glucose tolerance test data were not available; therefore, glycemic status could not be comprehensively established using standard biochemical diagnostic criteria. The primary outcomes were the estimated diabetes-risk classifications derived from FINDRISC, QDScore, and CANRISK.

### 2.4. Assessment of Type 2 Diabetes Risk

Diabetes risk was estimated using three validated screening tools: the Finnish Diabetes Risk Score (FINDRISC) [52], the Q Diabetes Risk Score (QDScore) [53], and the Canadian Diabetes Risk Questionnaire (CANRISK) [54]. The three risk instruments were calculated using IBM SPSS Statistics version 31.0 (IBM Corp., Armonk, NY, USA). The three risk instruments were originally developed or validated in populations with different age ranges. FINDRISC was originally derived in adults aged 35–64 years, whereas QDScore was developed and validated in individuals aged 25–79 years. CANRISK was originally developed primarily for adults aged ≥40 years, although its performance has subsequently been evaluated in younger adults. Consequently, the full age range of the present cohort (18–69 years) does not fall within the original derivation or validation age ranges of all three instruments. Nevertheless, these tools have previously been applied concurrently in a large Spanish occupational cohort of workers aged 18–69 years [55]. Their use in the present study therefore allows comparative risk stratification across instruments, but should not be interpreted as evidence that each score has been formally validated or recalibrated across the entire age range of Spanish workers. These instruments assess the probability of developing type 2 diabetes based on different combinations of age, sex, body mass index (BMI), waist circumference, blood pressure treatment, dietary habits, physical activity, family history of diabetes, and, in the case of QDScore and CANRISK, additional sociodemographic and clinical variables. Participants were categorized into low-, moderate-, or high-risk groups based on each tool’s recommended cut-off values. The applicability of these risk tools to the present occupational cohort requires consideration of both age range and population context. The study population comprised Spanish workers aged 18–69 years. Although the three instruments were originally developed and validated in different populations and their original validation age ranges are not identical, these risk scores have previously been applied concurrently in a large Spanish occupational cohort of 383,980 workers aged 18–69 years [55]. This previous study supports their feasibility and comparative use for diabetes risk stratification in Spanish working populations. However, this application should not be considered equivalent to formal external validation or recalibration of each instrument specifically for Spanish workers. Accordingly, the scores are used in the present study as established risk-classification tools, and comparisons between instruments should be interpreted in light of differences in their original derivation populations and age specifications. Because these diabetes risk scores are composite instruments that incorporate demographic, anthropometric, clinical, and, depending on the instrument, lifestyle-related components, some variables examined as correlates in the present study may also contribute directly or indirectly to the construction of the corresponding risk score. Therefore, associations involving variables that are components of a given score should be interpreted partly as reflecting the structure of the risk algorithm rather than as fully independent associations with diabetes risk.

### 2.5. Burnout Assessment

Occupational burnout was assessed using the Burnout Assessment Tool (BAT), a 23-item instrument designed to evaluate four core dimensions of burnout: exhaustion, mental distance, cognitive impairment, and emotional impairment [56]. BAT items are rated on a 5-point Likert scale ranging from 1 (never) to 5 (always). In the occupational health database used for the present study, burnout was available exclusively as a previously derived categorical variable with three levels (low, moderate, and high). Individual item responses, domain-specific scores, continuous global BAT scores, and the underlying scoring information used to derive these categories were not available in the analytical dataset. Consequently, the present analyses used the predefined low, moderate, and high burnout categories as provided by the occupational health database, and the original continuous BAT scores or domain-specific classifications could not be reconstructed or independently verified.

### 2.6. Lifestyle Variables

Adherence to a healthy dietary pattern was evaluated using the 14-item Mediterranean Diet Adherence Screener (MEDAS-14), which assesses the frequency of intake of key components of the Mediterranean diet such as olive oil, fruits, vegetables, legumes, and fish, as well as unhealthy items such as red meat and sugary beverages [57]. A MEDAS-14 score ≥ 9 was considered indicative of good adherence to the Mediterranean diet.

Physical activity was assessed using the International Physical Activity Questionnaire—Short Form (IPAQ-SF), which captures self-reported physical activity over the last seven days, including walking, moderate and vigorous activities, and time spent sitting [58]. Based on scoring guidelines, participants were categorized into low, moderate, or high physical activity levels.

Smoking status was classified as current smoker or non-smoker based on self-report.

### 2.7. Sociodemographic and Occupational Variables

Sociodemographic variables included age, sex, and occupational social class. Occupational social class was classified according to the Spanish Society of Epidemiology classification system into three categories (I–III), with Class I representing the highest socioeconomic group [59].

### 2.8. Statistical Analysis

Descriptive analyses were conducted to summarize the characteristics of the study population, stratified by sex. Continuous variables were expressed as means and standard deviations, and categorical variables as absolute and relative frequencies. Comparisons between groups were performed using Student’s t-test for continuous variables and the chi-square test for categorical variables.

Multivariable logistic regression models were constructed to identify independent associations between sociodemographic, lifestyle, and psychosocial factors (including burnout levels) and the probability of being classified as moderate-to-high diabetes risk, based on each of the three screening tools. Odds ratios (ORs) and 95% confidence intervals (CIs) were calculated. Tests for linear trend across burnout categories were performed by modeling burnout as an ordinal variable. Predictor–outcome overlap was considered when interpreting the multivariable models. Associations between sociodemographic or lifestyle variables and a diabetes risk score were not interpreted as independent predictive effects when the corresponding variable formed part of, or closely overlapped with, the construction of that score. Burnout, which is not a component of FINDRISC, QDScore, or CANRISK, was considered the primary exposure of interest.

Mediation Analysis: To explore potential behavioral pathways linking burnout to type 2 diabetes risk, mediation analyses were performed to evaluate whether physical inactivity (IPAQ-SF category), and low adherence to the Mediterranean diet (MEDAS-14 score < 9) mediated the association between burnout levels and moderate-to-high diabetes risk.

Burnout was modeled as the independent variable, diabetes risk (moderate-to-high category according to FINDRISC, QDScore, and CANRISK separately) as the dependent variable, and physical activity and Mediterranean diet adherence as parallel mediators.

Analyses were conducted using PROCESS macro (Model 4) with 5000 bootstrap resamples to estimate indirect effects and 95% bias-corrected confidence intervals. Age, sex, social class, and smoking status were included as covariates. Mediation was considered significant when the confidence interval of the indirect effect did not include zero. The proportion of the total effect mediated was calculated. Direct effects were examined to distinguish between partial and complete mediation. Given the cross-sectional design, temporal ordering between burnout, lifestyle behaviors, and estimated diabetes risk could not be established. Therefore, the mediation analyses were considered exploratory and the indirect effects were interpreted as statistical associations compatible with the hypothesized pathways rather than as evidence of causal mediation. In addition, because physical activity contributes directly to the construction of some of the diabetes risk algorithms, the indirect association involving physical inactivity may partly reflect mathematical overlap between the proposed mediator and the outcome. Accordingly, this pathway should be interpreted with particular caution and regarded as exploratory.

Predictive performance and incremental value of burnout: The incremental contribution of burnout to the identification of individuals classified at moderate-to-high type 2 diabetes risk was evaluated using discrimination and reclassification metrics. For each risk tool (FINDRISC, QDScore, and CANRISK), we fitted a base multivariable model including age, sex, social class, smoking status, Mediterranean diet adherence (MEDAS-14), and physical activity (IPAQ-SF). We then fitted an extended model additionally including burnout (BAT categories).

Model discrimination was quantified using the area under the receiver operating characteristic curve (AUC) with 95% confidence intervals. Differences in AUC between the base and extended models were assessed using DeLong’s test. The incremental predictive value of burnout was further evaluated using the continuous (category-free) net reclassification improvement (NRI) and the integrated discrimination improvement (IDI). Statistical significance was defined as a two-sided *p* value < 0.05.

Interaction analyses: To assess potential effect modification, multiplicative interaction terms were introduced into the fully adjusted logistic regression models to evaluate whether the association between burnout and moderate-to-high diabetes risk differed by sex, physical activity (IPAQ-SF), and social class. Separate models including burnout × sex, burnout × physical activity, and burnout × social class interaction terms were fitted for each diabetes risk tool. Significant interactions were further explored through stratified analyses, and interaction effects were considered statistically significant at *p* < 0.05.

Sensitivity analyses: Sensitivity analyses were conducted to evaluate the robustness of the main findings by additionally adjusting models for body mass index and by excluding participants aged ≥60 years and those with severe obesity (BMI ≥ 35 kg/m^2^). Associations, mediation effects, and predictive performance metrics were reassessed in these sensitivity models. To address potential differences between the age range of the study population and the original derivation or validation ranges of the diabetes risk instruments, an additional sensitivity analysis was performed restricting the sample to participants aged 40–64 years.

All statistical analyses were performed using IBM SPSS Statistics version 31.0 (IBM Corp., Armonk, NY, USA). Statistical significance was defined as a two-sided *p*-value < 0.05.

## 3. Results

Table 1 summarizes the sociodemographic, anthropometric, clinical, lifestyle, and burnout characteristics of the study population stratified by sex. Significant sex differences were observed across all variables (all *p* < 0.001). Men were older, taller, and heavier and had higher waist circumference, blood pressure, triglyceride, and glucose levels, whereas women had higher HDL cholesterol concentrations and were more likely to report adherence to the Mediterranean diet and regular physical activity. The prevalence of high burnout was also lower among women than among men.

Table 2 shows the prevalence of moderate-to-high type 2 diabetes risk according to FINDRISC, QDScore, and CANRISK across sociodemographic characteristics, lifestyle behaviors, and burnout categories. The prevalence of moderate-to-high diabetes risk increased progressively with age and was consistently higher among individuals from lower social classes, current smokers, those with poor adherence to the Mediterranean diet, physically inactive participants, and those with higher burnout levels (all *p* < 0.001).

Table 3 presents the adjusted odds ratios (ORs) and 95% confidence intervals (CIs) for moderate-to-high diabetes risk according to the three screening tools. Male sex, older age, lower social class, current smoking, non-adherence to the Mediterranean diet, physical inactivity, and higher burnout levels were associated with increased odds of being classified as having moderate-to-high diabetes risk across the three models. For variables that are components of, or overlap with, the corresponding risk algorithms, these associations should be interpreted in the context of predictor–outcome overlap rather than as independent predictive effects. In contrast, burnout is not included in the construction of FINDRISC, QDScore, or CANRISK. A significant dose–response association was observed across increasing burnout categories (*p* for trend < 0.001).

**Figure 2:**
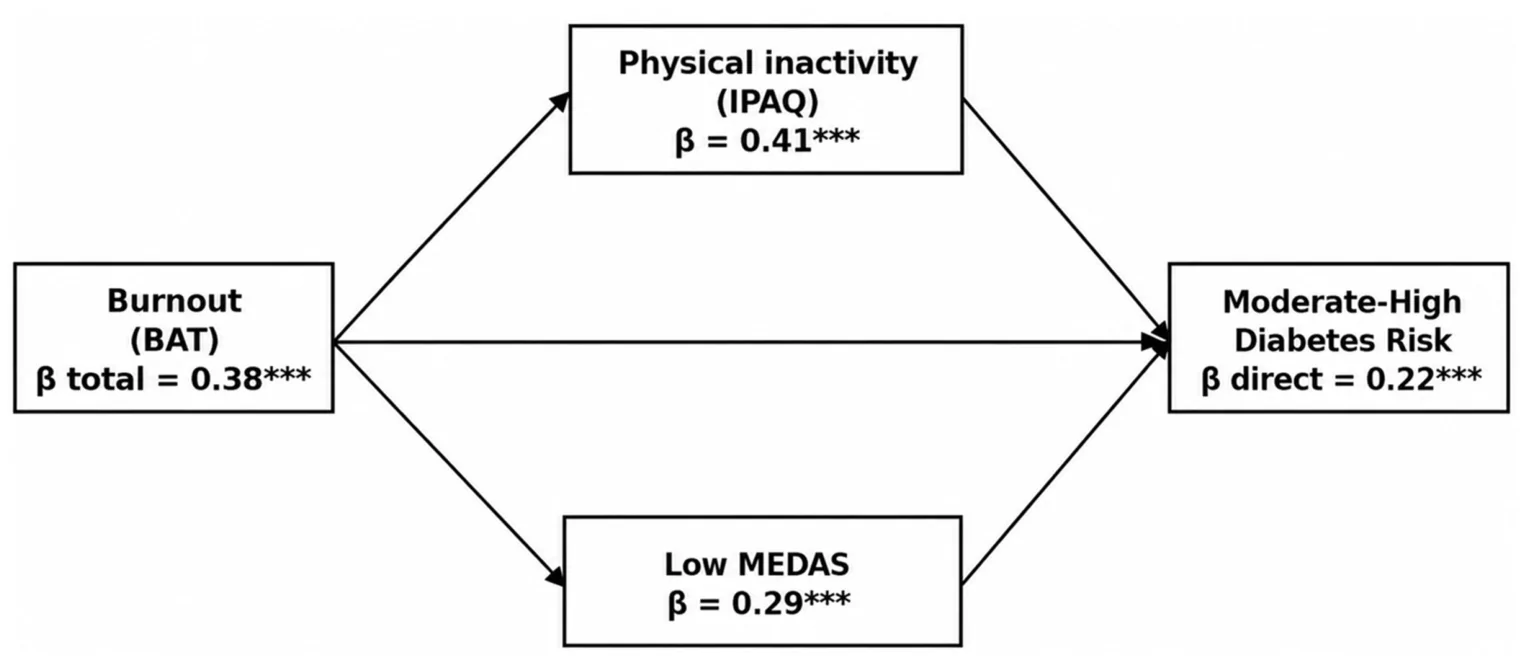
Parallel mediation model of the association between burnout and moderate-to-high type 2 diabetes risk according to FINDRISC. Physical inactivity and low adherence to the Mediterranean diet significantly mediated the association between burnout and moderate-to-high diabetes risk after adjustment for age, sex, social class, and smoking status. Together, these lifestyle behaviors accounted for approximately 45% of the total association, with physical inactivity showing the largest mediated effect. The persistence of a statistically significant direct association after inclusion of both lifestyle variables is compatible with partial statistical mediation. However, because burnout, lifestyle behaviors, and estimated diabetes risk were assessed cross-sectionally, these indirect associations do not establish temporal or causal mediation. Moreover, because physical activity contributes directly to the construction of some diabetes risk algorithms, the indirect pathway involving physical inactivity should be interpreted with additional caution because of potential mediator–outcome overlap. β, standardized regression coefficient; *** *p* < 0.001.

Physical inactivity and low adherence to the Mediterranean diet showed significant indirect associations in the relationship between burnout and moderate-to-high diabetes risk after adjustment for age, sex, social class, and smoking status (Figure 2). Bootstrap analyses (5000 resamples) demonstrated statistically significant indirect associations for both lifestyle variables, which together accounted for approximately 45% of the total statistical association between burnout and diabetes risk. Physical inactivity accounted for the largest proportion of this indirect association. The direct association between burnout and diabetes risk remained statistically significant after the inclusion of both lifestyle variables. Comparable patterns were observed when diabetes risk was assessed using QDScore and CANRISK (Supplementary Figures S1 and S2). Given the cross-sectional design, these findings should be interpreted as statistical indirect associations compatible with the hypothesized behavioral pathways and not as evidence of temporal or causal mediation. The indirect association involving physical inactivity warrants additional caution because physical activity contributes to the construction of some of the diabetes risk algorithms and may therefore partly reflect mediator–outcome overlap.

**Figure 3:**
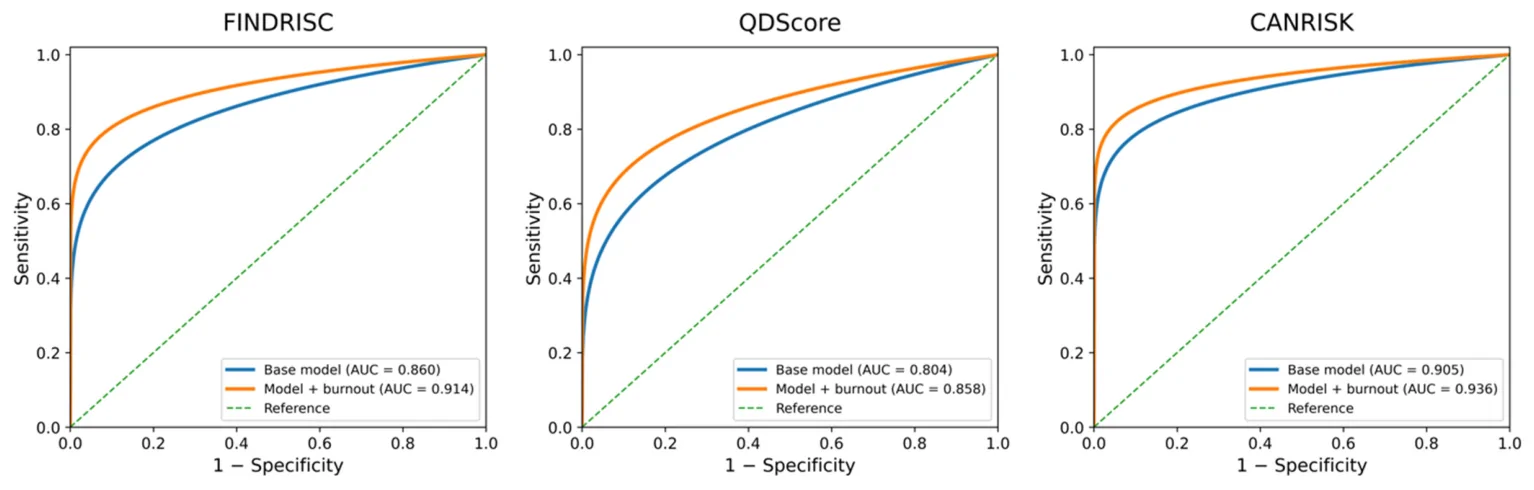
ROC curves comparing the base multivariable model with the extended model including burnout for the identification of moderate-to-high type 2 diabetes risk according to FINDRISC, QDScore, and CANRISK. The base model included age, sex, social class, smoking status, Mediterranean diet adherence, and physical activity, whereas the extended model additionally included burnout. Adding burnout significantly improved discrimination across all three risk tools, increasing the AUC from 0.860 to 0.914 for FINDRISC, from 0.804 to 0.858 for QDScore, and from 0.905 to 0.936 for CANRISK (DeLong *p* < 0.001 for all comparisons). The greater AUC of the extended models indicates improved discrimination between participants classified as having moderate-to-high versus lower estimated diabetes risk according to the corresponding risk scores; these analyses do not assess prediction of incident T2DM.

Incremental discrimination and reclassification associated with burnout. Incorporation of burnout into the base multivariable models significantly improved discrimination for identifying individuals classified as having moderate-to-high diabetes risk according to all three screening tools (Table 4 and Figure 3). For FINDRISC, the AUC increased from 0.860 (95% CI 0.856–0.863) to 0.914 (95% CI 0.911–0.916), corresponding to a ΔAUC of 0.054 (*p* < 0.001). For QDScore, the AUC increased from 0.804 (95% CI 0.801–0.808) to 0.858 (95% CI 0.855–0.861; ΔAUC = 0.054, *p* < 0.001). For CANRISK, the AUC increased from 0.905 (95% CI 0.902–0.907) to 0.936 (95% CI 0.934–0.938; ΔAUC = 0.031, *p* < 0.001).

Category-free reclassification analyses further demonstrated the incremental contribution of burnout to discrimination and reclassification of moderate-to-high diabetes-risk classification across all three risk tools. Continuous NRI values were 1.022 for FINDRISC, 0.419 for QDScore, and 1.210 for CANRISK, indicating significant improvements in risk reclassification after the inclusion of burnout. Similarly, IDI values were 0.103, 0.058, and 0.084, respectively, demonstrating improved discrimination for identifying individuals with moderate-to-high type 2 diabetes risk (all *p* < 0.001).

**Figure 4:**
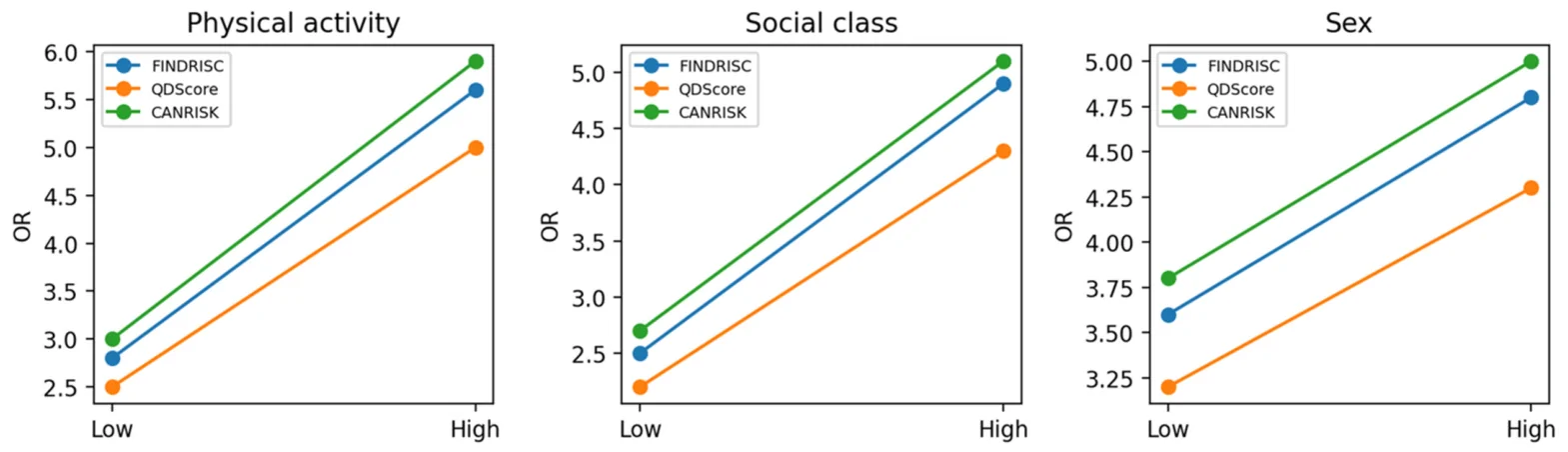
Stratified odds ratios for the association between high burnout and moderate-to-high type 2 diabetes risk according to physical activity, social class, and sex. Significant effect modification was observed across these subgroups. The association between high burnout and diabetes risk was stronger among physically inactive participants and individuals from lower social classes, with a more modest but significant difference according to sex, showing higher effect estimates in men. These findings suggest that the association between burnout and diabetes risk may be particularly pronounced in socially and behaviorally vulnerable groups.

Interaction analyses: Significant effect modification was observed for the association between burnout and moderate-to-high diabetes risk according to physical activity, social class, and sex (all *p*-interaction < 0.05; Table 5, Figure 4). The association between high burnout and moderate-to-high diabetes risk was stronger among physically inactive individuals (OR = 5.6) than among physically active participants (OR = 2.8). Similarly, the association was stronger in participants from lower social classes (OR = 4.9 in Class III vs. OR = 2.5 in Class I). A modest but statistically significant interaction with sex was also observed, with higher effect estimates in men than in women.

Sensitivity analyses: The results remained materially unchanged after additional adjustment for body mass index and after excluding participants aged ≥60 years and those with severe obesity (BMI ≥ 35 kg/m^2^). The associations between burnout and moderate-to-high diabetes risk, the magnitude of the mediation effects, and the incremental discrimination and reclassification associated with burnout (ΔAUC, continuous NRI, and IDI) remained consistent across these sensitivity analyses. In an additional analysis restricted to participants aged 40–64 years (*n* = 44,372), the association between burnout and moderate-to-high estimated diabetes risk remained significant across all three instruments. Compared with low burnout, moderate burnout was associated with ORs of 6.63 (95% CI 5.04–8.72) for FINDRISC, 1.91 (95% CI 1.65–2.21) for QDScore, and 2.17 (95% CI 2.03–2.32) for CANRISK, whereas high burnout was associated with ORs of 44.78 (95% CI 34.29–58.47), 6.17 (95% CI 5.39–7.06), and 6.99 (95% CI 6.48–7.54), respectively (all *p* < 0.001). These findings support the robustness of the association between burnout and estimated diabetes-risk classification when the analysis is restricted to a narrower age range compatible with the principal age ranges of the three instruments.

The combined forest plot provides a visual summary of the adjusted odds ratios (ORs) and 95% confidence intervals (CIs) for moderate-to-high diabetes risk, as estimated by three established diabetes risk assessment tools—FINDRISC, QDScore, and CANRISK—across key sociodemographic, lifestyle, and psychosocial variables.

Sex Differences: Men showed greater odds of being classified as having moderate-to-high diabetes risk across the three screening tools. Because sex is incorporated into, or contributes to, the construction of some of the corresponding risk algorithms, these estimates should not be interpreted as independent predictive effects.

Age Gradient: A steep and consistent increase in the odds of being classified as having moderate-to-high diabetes risk was observed with increasing age across the three screening tools. However, because age is incorporated into the construction of these risk scores, these associations should be interpreted primarily as reflecting the structure of the corresponding risk algorithms rather than as independent predictive effects.

Social Class: Compared with individuals in social class I, those in lower classes showed elevated odds of diabetes risk, particularly under the CANRISK model (OR = 2.63 for class III), suggesting that socioeconomic disadvantage is associated with poorer metabolic health, potentially mediated by lifestyle and access to healthcare.

Lifestyle Factors: Smoking, non-adherence to the Mediterranean diet, and physical inactivity were associated with greater odds of being classified as having moderate-to-high diabetes risk. However, because lifestyle-related variables are incorporated to varying degrees into the diabetes risk algorithms, particularly physical activity and dietary components, the magnitude of these associations should be interpreted cautiously and not as independent predictive effects.

Burnout: Psychosocial stress, measured via burnout levels, also exhibited a dose–response association with diabetes risk. Individuals with high burnout had markedly elevated odds, especially in Findrisc (OR = 4.62) and QDScore (OR = 4.61), reinforcing the link between mental health and metabolic dysregulation.

Interpretation Across Tools: Although all three instruments identified similar patterns, some differences in effect magnitude are notable:

- CANRISK consistently showed higher ORs for sex and age categories, reflecting its broader inclusion of family history and lifestyle elements.
- QDScore appeared more sensitive to lifestyle and burnout variables.
- Findrisc, while consistent, provided slightly lower ORs, possibly due to its narrower item scope.

**Figure 5:**
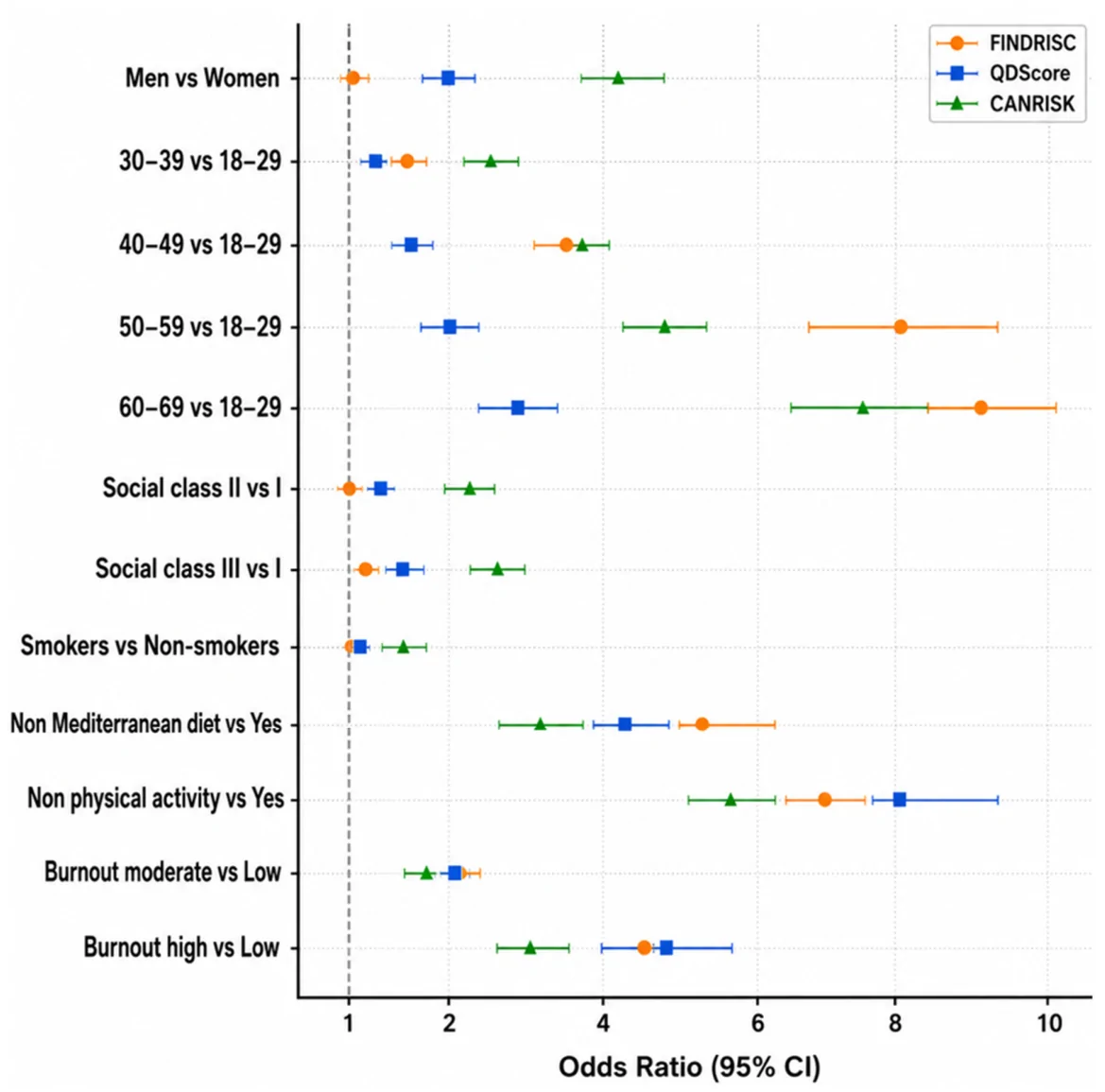
Forest plot of adjusted odds ratios (ORs) and 95% confidence intervals (CIs) for moderate-to-high type 2 diabetes risk across FINDRISC, QDScore, and CANRISK. The models show a consistent pattern across the three risk assessment tools, with higher odds of moderate-to-high diabetes risk associated with older age, male sex, lower social class, smoking, physical inactivity, low adherence to the Mediterranean diet, and higher burnout levels. Particularly strong associations were observed for older age, physical inactivity, low Mediterranean diet adherence, and high burnout. Burnout showed a dose–response pattern, with progressively higher odds of diabetes risk at increasing burnout levels. Although the overall direction of associations was broadly consistent across FINDRISC, QDScore, and CANRISK, estimates for variables that contribute to the corresponding risk algorithms should be interpreted in light of potential predictor–outcome overlap. In contrast, occupational burnout is not included in any of the three risk scores, making its consistent association across the three instruments particularly noteworthy.

The forest plot shows broadly consistent patterns of association across the three diabetes risk tools. However, associations involving variables that contribute to the construction of the corresponding risk scores should be interpreted in light of this predictor–outcome overlap. In contrast, occupational burnout is not included in the construction of FINDRISC, QDScore, or CANRISK, making its consistent association with moderate-to-high estimated diabetes risk across all three instruments particularly noteworthy (Figure 5).

### Summary of Key Findings

Taken together, these results indicate that burnout is consistently associated with elevated estimated diabetes risk and that physical inactivity and low adherence to the Mediterranean diet statistically account for part of this cross-sectional association. These indirect associations are compatible with the hypothesized behavioral pathways but do not establish temporal or causal mediation. Moreover, incorporation of burnout significantly improved the discrimination and reclassification of moderate-to-high estimated diabetes risk across the three screening tools; however, these findings refer to classification based on existing risk scores and not to the prediction of incident T2DM, and the association between burnout and estimated diabetes risk was stronger among socially and behaviorally vulnerable groups. These converging findings support the relevance of burnout as a psychosocial factor associated with metabolic risk in working populations.

## 4. Discussion

### 4.1. Principal Findings and Context

This large cross-sectional study provides further evidence of an association between burnout and moderate-to-high type 2 diabetes risk as assessed using three validated screening tools (FINDRISC, QDScore, and CANRISK). High burnout levels were consistently associated with greater odds of being classified as having moderate-to-high diabetes risk across the three screening tools. Associations observed for sociodemographic and lifestyle variables should be interpreted cautiously because some of these variables are incorporated into, or overlap with, the construction of the corresponding diabetes risk algorithms. These findings are consistent with and extend previous evidence on the associations between biopsychosocial factors and cardiometabolic health.

### 4.2. Comparison with Previous Studies

Burnout, a state of emotional, physical, and mental exhaustion caused by prolonged occupational stress, has increasingly been recognized as a risk factor for metabolic disorders, including insulin resistance and T2DM. Previous longitudinal studies have reported that individuals experiencing high burnout symptoms have significantly increased odds of incident diabetes. For example, Melamed et al. found a 1.8-fold higher risk of T2DM in individuals with high burnout in an Israeli cohort [60]. Similarly, two studies indicated that job strain was associated with elevated fasting glucose and HbA1c levels, independent of body weight and other confounders [61,62].

Psychoneuroendocrine mechanisms may partly explain this relationship, particularly through chronic activation of the hypothalamic–pituitary–adrenal (HPA) axis, elevated cortisol levels, inflammation, and autonomic dysfunction [63,64]. A recent prospective observational study in Italian nurses supported the association between work-related stress and metabolic syndrome, with burnout being a central mediator [65]. These pathophysiological pathways overlap with those involved in T2DM development and provide biological plausibility for the observed association.

Despite this, few large occupational studies have simultaneously assessed burnout and diabetes risk using validated tools. One exception is a Chinese study which found that burnout was significantly associated with increased insulin resistance and metabolic syndrome markers among healthcare workers [66]. Our findings extend previous evidence by demonstrating consistent associations across a large and diverse occupational cohort while incorporating detailed sociodemographic and behavioral characterization.

### 4.3. Contributions and Implications

In the exploratory cross-sectional mediation analyses, physical inactivity and low adherence to the Mediterranean diet statistically accounted for approximately 45% of the observed association between burnout and estimated diabetes risk. These indirect associations are compatible with the hypothesized behavioral pathways but should not be interpreted as evidence of temporal or causal mediation. Furthermore, the pathway involving physical inactivity requires particular caution because physical activity contributes directly to some of the diabetes risk algorithms, potentially introducing mediator–outcome overlap. The observed improvements in discrimination and reclassification indicate that burnout provides additional information for distinguishing between individuals classified at lower versus moderate-to-high estimated diabetes risk by the existing screening tools. However, because the outcomes were derived from cross-sectional risk-score classifications rather than incident T2DM, these findings should not be interpreted as evidence that burnout improves the prediction of future diabetes.

This study makes several novel contributions. First, it is one of the few to examine the relationship between burnout and T2DM risk using a large, heterogeneous occupational cohort. Second, it utilizes three internationally recognized screening tools for diabetes risk, providing consistent and comparable estimates across subpopulations. Third, it integrates the assessment of burnout using the validated Burnout Assessment Tool, enabling standardized evaluation and categorization.

The observed association between burnout and estimated diabetes risk suggests that psychosocial factors may warrant consideration alongside traditional behavioral and sociodemographic factors when characterizing cardiometabolic risk in occupational populations. However, given the cross-sectional design and the use of diabetes risk-score classifications rather than incident T2DM outcomes, these findings do not establish the clinical effectiveness of routine burnout screening for diabetes prevention. Prospective and intervention studies are needed to determine whether incorporating burnout assessment into occupational health strategies improves the identification of individuals at future risk of T2DM or contributes to improved cardiometabolic outcomes.

### 4.4. Future Directions

The findings highlight several important directions for future research. First, prospective studies are needed to confirm the temporal relationship and clarify potential causal pathways between burnout and T2DM development. Second, intervention studies should assess whether workplace-based mental health programs and stress management strategies can reduce diabetes incidence among at-risk employees. Third, the integration of digital health monitoring tools, such as wearable devices and mobile apps, could facilitate longitudinal tracking of physiological and psychological indicators, enhancing early detection efforts [67].

Furthermore, research should explore sex-specific and occupational-sector-specific differences in the burnout–diabetes risk association, given the potential for differential exposure and vulnerability. Future studies integrating biomarkers (e.g., cortisol, inflammatory markers, and HOMA-IR) may further elucidate the biological mechanisms underlying the observed associations.

### 4.5. Strengths and Limitations

This study has multiple strengths. The large sample size (*n* = 92.480) drawn from diverse economic sectors provides substantial statistical power and enhances generalizability to working populations. The use of three validated T2DM risk scales allows for triangulation and consistency in outcomes. Burnout was assessed using the Burnout Assessment Tool (BAT), providing a standardized assessment of work-related burnout. The comprehensive assessment of lifestyle variables using validated instruments (MEDAS-14 and IPAQ-SF), together with the standardized classification of occupational social class, further strengthens the methodological rigor of the study.

However, several limitations must be acknowledged. The cross-sectional design precludes causal inference and does not allow the temporal ordering required for causal mediation to be established. Consequently, the mediation findings should be interpreted as exploratory statistical indirect associations rather than as evidence that physical inactivity or low adherence to the Mediterranean diet causally mediate the association between burnout and estimated diabetes risk. Reverse or bidirectional relationships between burnout, lifestyle behaviors, and estimated diabetes risk cannot be excluded. Moreover, because physical activity is incorporated into some of the diabetes risk algorithms, the indirect association observed through physical inactivity may be partly influenced by mediator–outcome overlap and should therefore be interpreted with additional caution. Although multiple confounders were adjusted for, residual confounding cannot be excluded. All measures, including burnout, lifestyle habits, and medical history, relied on self-report, which may introduce recall or social desirability bias. Regarding burnout assessment, the analytical database contained only the predefined BAT categories (low, moderate, and high), whereas individual item responses and continuous BAT scores were not available for analysis. Therefore, the individual BAT dimensions could not be examined separately. Although fasting plasma glucose and lipid measurements were available from the occupational health examinations, HbA1c and oral glucose tolerance test data were not available. Consequently, glycemic status could not be comprehensively characterized using standard biochemical diagnostic criteria, and fasting glucose was therefore used only for descriptive characterization of the study population rather than to define diabetes or prediabetes outcomes. The primary outcomes of the present study were the estimated diabetes-risk classifications derived from FINDRISC, QDScore, and CANRISK, rather than biochemically confirmed glycemic status. Furthermore, the discrimination and reclassification analyses used moderate-to-high risk classifications derived from FINDRISC, QDScore, and CANRISK as outcomes rather than incident T2DM. Therefore, the observed improvements in AUC, NRI, and IDI after inclusion of burnout indicate improved discrimination and reclassification of risk-score categories within this cross-sectional dataset and should not be interpreted as evidence of improved prediction of future T2DM. Prospective studies using incident T2DM as the outcome are required to determine whether burnout provides incremental predictive value for future diabetes. An additional methodological consideration is the potential overlap between some predictors and the components used to construct the diabetes risk scores. Variables such as age, sex, anthropometric characteristics, and certain lifestyle-related factors are incorporated, to varying degrees, into FINDRISC, QDScore, and CANRISK. Consequently, associations observed between these variables and moderate-to-high estimated diabetes risk may partly reflect the mathematical structure of the corresponding risk algorithms and should not be interpreted as independent validation of their relationship with diabetes risk. Importantly, occupational burnout is not included as a component of any of the three risk scores, reducing the potential for direct predictor–outcome circularity for the primary exposure of interest. A further limitation concerns the applicability of the diabetes risk instruments to the age and occupational context of the study population. The original derivation or validation age ranges of FINDRISC, QDScore, and CANRISK do not fully encompass the entire 18–69-year age range included in this study. Moreover, although these instruments have previously been applied in large Spanish occupational populations, they have not been specifically recalibrated for the present cohort. Therefore, absolute risk estimates and between-tool differences should be interpreted cautiously, particularly in participants outside the original age ranges of the respective instruments. Finally, although the BAT is a validated instrument for assessing burnout, the analytical dataset contained burnout as a predefined categorical variable rather than the original item-level or continuous scores, precluding analyses based on continuous BAT scores or individual dimensions [55].

## 5. Conclusions

This study provides further evidence that burnout, an increasingly prevalent occupational health concern, is independently associated with moderate-to-high type 2 diabetes risk, as assessed by three validated risk scores in a large and diverse working population. The integration of sociodemographic, lifestyle, and mental health variables reveals the complex interplay of factors contributing to cardiometabolic vulnerability in the occupational setting.

The findings suggest that psychosocial factors such as burnout may provide additional information when characterizing individuals with elevated estimated diabetes risk in occupational populations. However, the cross-sectional nature of the study and the use of risk-score classifications rather than incident T2DM outcomes preclude recommendations regarding the routine incorporation of burnout assessment into diabetes screening protocols. Prospective studies are needed to determine the clinical utility of such an approach.

The observed associations also highlight the relevance of lifestyle and socioeconomic factors in the relationship between burnout and estimated diabetes risk. In particular, physical inactivity, lower adherence to the Mediterranean diet, and socioeconomic disadvantage were associated with less favorable risk profiles. These findings may help inform hypotheses for future prospective and intervention studies examining whether combined psychosocial and lifestyle approaches can influence subsequent cardiometabolic outcomes in working populations.

Taken together, these findings highlight an association between psychosocial, lifestyle, and estimated metabolic risk factors in working populations. They provide a basis for further prospective research to determine whether incorporating psychosocial factors into broader chronic disease prevention frameworks may offer additional value beyond established risk factors.

Future longitudinal studies are warranted to confirm the temporal relationship between burnout and diabetes development, and to evaluate the effectiveness of preventive measures. Such studies will be necessary to determine whether burnout assessment has clinical or public health utility for identifying individuals at future risk of T2DM and whether interventions targeting psychosocial factors can contribute to diabetes prevention.

## Figures and Tables

**Table 1:** Baseline characteristics of the study population according to sex (*n* = 92,480).

Variable	Total (*n* = 92,480)	Men (*n* = 55,918)	Women (*n* = 36,562)	*p*-Value
Continuous variables, mean (SD)				
Age (years)	39.5 (10.3)	39.8 (10.4)	39.1 (10.1)	<0.001
Height (cm)	168.9 (9.3)	174.0 (7.1)	161.2 (6.6)	<0.001
Weight (kg)	75.0 (15.6)	81.1 (13.8)	65.5 (13.3)	<0.001
Waist circumference (cm)	82.3 (11.0)	87.7 (9.2)	74.0 (7.9)	<0.001
Hip circumference (cm)	98.9 (8.7)	100.1 (8.4)	97.2 (9.0)	<0.001
Systolic blood pressure (mmHg)	120.4 (15.7)	124.4 (15.1)	114.2 (14.6)	<0.001
Diastolic blood pressure (mmHg)	73.1 (10.8)	75.4 (10.7)	69.5 (10.2)	<0.001
Total cholesterol (mg/dL)	195.1 (37.9)	196.1 (38.7)	193.7 (36.7)	<0.001
HDL cholesterol (mg/dL)	52.1 (7.4)	51.0 (7.0)	53.7 (7.6)	<0.001
LDL cholesterol (mg/dL)	121.2 (37.4)	120.5 (37.4)	122.4 (37.3)	<0.001
Triglycerides (mg/dL)	110.0 (76.9)	124.3 (88.8)	88.1 (45.9)	<0.001
Fasting glucose (mg/dL)	86.5 (12.6)	88.1 (12.9)	84.1 (11.7)	<0.001
Categorical variables, n (%)				
Age group				<0.001
18–29 years	17,302 (18.7)	10,070 (18.0)	7232 (19.8)	
30–39 years	30,656 (33.1)	18,358 (32.8)	12,298 (33.6)	
40–49 years	27,264 (29.5)	16,532 (29.6)	10,732 (29.4)	
50–59 years	14,610 (15.8)	9186 (16.4)	5424 (14.8)	
60–69 years	2648 (2.9)	1772 (3.2)	876 (2.4)	
Social class				<0.001
Class I	5496 (5.9)	2982 (5.3)	2514 (6.9)	
Class II	21,974 (23.8)	9802 (17.5)	12,172 (33.3)	
Class III	65,010 (70.3)	43,134 (77.1)	21,876 (59.8)	
Current smokers	32,538 (35.2)	20,708 (37.0)	11,830 (32.4)	<0.001
Mediterranean diet adherence	41,670 (45.1)	22,880 (40.9)	18,790 (51.4)	<0.001
Physically active	44,538 (48.2)	25,534 (45.7)	19,004 (52.0)	<0.001
Burnout level				<0.001
Low	38,820 (42.0)	20,584 (36.8)	18,236 (49.9)	
Moderate	29,672 (32.1)	18,326 (32.8)	11,346 (31.0)	
High	23,988 (25.9)	17,008 (30.4)	6980 (19.1)	

BP, blood pressure. HDL, high-density lipoprotein. LDL, low-density lipoprotein. SD, standard deviation.

**Table 2:** Prevalence of moderate-to-high type 2 diabetes risk according to sociodemographic characteristics, lifestyle behaviours and burnout, stratified by sex.

Men (*n* = 55,918)				
Characteristic	*n*	FINDRISC (%)	QDScore ≥ 3 (%)	CANRISK High (%)
Age group				
18–29 years	10,070	1.4	5.7	1.4
30–39 years	18,358	2.5	7.3	2.6
40–49 years	16,532	9.7	9.8	11.2
50–59 years	9186	22.4	10.8	33.2
60–69 years	1772	30.0	11.6	49.7
Social class				
Class I	2982	6.5	7.4	5.5
Class II	9802	8.3	7.5	7.1
Class III	43,134	8.8	9.2	12.9
Smoking				
Yes	20,708	9.2	9.5	12.4
No	35,210	7.5	8.3	9.8
Mediterranean diet adherence				
Yes	22,880	3.8	4.8	5.3
No	33,038	9.6	9.2	12.6
Physical activity				
Yes	25,534	3.3	3.7	4.0
No	30,384	11.7	11.7	15.9
Burnout level				
Low	20,584	3.8	3.3	2.8
Moderate	18,326	5.7	6.9	7.6
High	17,008	15.8	14.6	16.7
Women (*n* = 36,562)				
Characteristic	*n*	FINDRISC (%)	QDScore ≥ 3 (%)	CANRISK High (%)
Age group				
18–29 years	7232	2.0	8.8	0.2
30–39 years	12,298	2.5	9.6	0.4
40–49 years	10,732	6.9	11.6	3.2
50–59 years	5424	16.6	13.0	11.2
60–69 years	876	29.0	13.8	22.8
Social class				
Class I	2514	3.8	6.1	0.7
Class II	12,172	4.1	7.5	1.0
Class III	21,876	8.0	13.4	4.9
Smoking				
Yes	11,830	7.1	11.1	4.0
No	24,732	4.9	10.4	1.8
Mediterranean diet adherence				
Yes	18,790	3.2	6.3	1.9
No	17,772	8.7	14.2	4.2
Physical activity				
Yes	19,004	2.5	5.1	1.3
No	17,558	9.8	17.2	6.0
Burnout level				
Low	18,236	3.9	3.9	2.1
Moderate	11,346	8.8	13.8	4.5
High	6980	14.2	23.6	8.7

Findrisc, Finnish Diabetes Risk Score. QDScore, Q Diabetes risk Score. CANRISK, Canadian Diabetes Risk Questionnaire. All comparisons between categories were statistically significant (χ^2^ test, *p* < 0.001).

**Table 3:** Adjusted multivariable logistic regression models for moderate-to-high type 2 diabetes risk according to FINDRISC, QDScore and CANRISK.

Variable	FINDRISC OR (95% CI)	*p*	QDScore OR (95% CI)	*p*	CANRISK OR (95% CI)	*p*
Sex						
Women (Ref)	1.00	—	1.00	—	1.00	—
Men	1.15 (1.11–1.19)	<0.001	2.11 (1.79–2.43)	<0.001	3.92 (3.64–4.21)	<0.001
Age group						
18–29 years (Ref)	1.00	—	1.00	—	1.00	—
30–39 years	1.63 (1.46–1.81)	<0.001	1.21 (1.16–1.26)	<0.001	2.42 (2.17–2.68)	<0.001
40–49 years	3.63 (3.25–4.02)	<0.001	1.50 (1.38–1.62)	<0.001	3.35 (3.08–3.62)	<0.001
50–59 years	7.92 (6.76–9.09)	<0.001	2.04 (1.74–2.34)	<0.001	4.49 (4.01–4.97)	<0.001
60–69 years	8.91 (7.87–9.96)	<0.001	2.88 (2.45–3.32)	<0.001	7.22 (6.31–8.12)	<0.001
Social class						
Class I (Ref)	1.00	—	1.00	—	1.00	—
Class II	1.15 (1.11–1.19)	<0.001	1.33 (1.25–1.42)	<0.001	2.15 (1.90–2.40)	<0.001
Class III	1.34 (1.25–1.44)	<0.001	1.56 (1.42–1.70)	<0.001	2.63 (2.41–2.86)	<0.001
Smoking						
No (Ref)	1.00	—	1.00	—	1.00	—
Yes	1.28 (1.20–1.36)	<0.001	1.16 (1.12–1.20)	<0.001	1.46 (1.37–1.56)	<0.001
Mediterranean diet adherence						
Yes (Ref)	1.00	—	1.00	—	1.00	—
No	5.24 (4.08–6.41)	<0.001	4.13 (3.24–5.03)	<0.001	3.02 (2.45–3.59)	<0.001
Physical activity						
Yes (Ref)	1.00	—	1.00	—	1.00	—
No	7.13 (6.01–8.26)	<0.001	7.83 (6.40–9.24)	<0.001	5.53 (4.86–6.20)	<0.001
Burnout						
Low (Ref)	1.00	—	1.00	—	1.00	—
Moderate	2.33 (2.08–2.59)	<0.001	2.13 (1.94–2.33)	<0.001	1.69 (1.54–1.75)	<0.001
High	4.62 (3.90–5.35)	<0.001	4.61 (3.66–5.56)	<0.001	2.89 (2.60–3.29)	<0.001

OR, odds ratio; CI, confidence interval; FINDRISC, Finnish Diabetes Risk Score; QDScore, QDiabetes Risk Score; CANRISK, Canadian Diabetes Risk Questionnaire. Models were adjusted for sex, age group, social class, smoking status, Mediterranean diet adherence, physical activity, and burnout level. Reference categories are indicated as (Ref).

**Table 4:** Incremental discrimination and reclassification associated with burnout for identifying moderate-to-high diabetes risk classification.

Outcome	Base Model AUC (95% CI)	Model + Burnout AUC (95% CI)	ΔAUC	DeLong *p*	Continuous NRI	NRI *p*	IDI	IDI *p*
FINDRISC	0.860 (0.856–0.863)	0.914 (0.911–0.916)	0.054	<0.001	1.022	<0.001	0.103	<0.001
QDScore	0.804 (0.801–0.808)	0.858 (0.855–0.861)	0.054	<0.001	0.419	<0.001	0.058	<0.001
CANRISK	0.905 (0.902–0.907)	0.936 (0.934–0.938)	0.031	<0.001	1.210	<0.001	0.084	<0.001

AUC, area under the receiver operating characteristic curve; CI, confidence interval; NRI, net reclassification improvement; IDI, integrated discrimination improvement; FINDRISC, Finnish Diabetes Risk Score; QDScore, QDiabetes Risk Score; CANRISK, Canadian Diabetes Risk Questionnaire. The base model included sex, age group, social class, smoking status, Mediterranean diet adherence, and physical activity. The extended model additionally included burnout level. Differences in AUC were evaluated using DeLong’s test. NRI was calculated using the category-free continuous method. All analyses were performed in the complete available sample for each outcome.

**Table 5:** Stratified associations between high burnout and moderate–high diabetes risk.

Outcome	IPAQ Inactive	Low Social Class	Men	*p*-Interaction
FINDRISC	5.6 (4.9–6.3)	4.9 (4.3–5.5)	4.8 (4.2–5.4)	<0.01
QDScore	5.0 (4.4–5.7)	4.3 (3.8–4.9)	4.3 (3.9–4.8)	<0.05
CANRISK	5.9 (5.1–6.7)	5.1 (4.5–5.8)	5.0 (4.4–5.6)	<0.01

## Data Availability

The original contributions presented in this study are included in the article/Supplementary Materials. Further inquiries can be directed to the corresponding author.

## Supplementary Materials

The following supporting information can be downloaded at: https://www.mdpi.com/article/10.3390/medsci14050546/s1, Figure S1: Parallel mediation model of the association between burnout and moderate-to-high type 2 diabetes risk according to QDScore; Figure S2. Parallel mediation model of the association between burnout and moderate-to-high type 2 diabetes risk according to CANRISK.
