# Supplementary material for: Lifestyle and Psychosocial Determinants of Type 2 Diabetes Risk Across Adulthood: The Role of Burnout in a Large Occupational Cohort
Source: Med Sci (Basel). 2026 Sep 3;14(5):546. doi: 10.3390/medsci14050546 (PMC13609176; doi:10.3390/medsci14050546)
Supplement: Supplementary file 1 [file medsci-14-00546-s001.zip › medsci-4501384-supplementary.pdf]

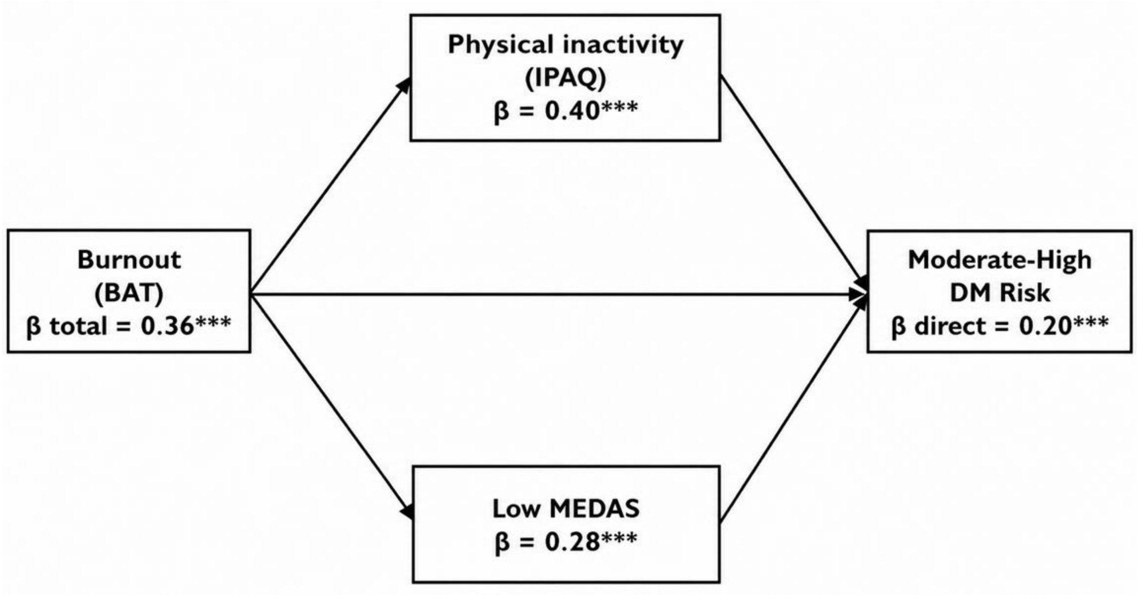

Supplementary Figure S1. Parallel mediation model of the association between burnout and moderate-to-high type 2 diabetes risk according to QDScore. Physical inactivity and low adherence to the Mediterranean diet were examined as parallel behavioral mediators of the association between burnout and diabetes risk, with adjustment for age, sex, social class, and smoking status. The figure displays the direct effect of burnout on diabetes risk and the indirect pathways operating through each lifestyle behavior. The persistence of a statistically significant direct association after inclusion of both lifestyle variables is compatible with partial statistical mediation. However, because burnout, lifestyle behaviors, and estimated diabetes risk were assessed cross-sectionally, these indirect associations do not establish temporal or causal mediation.

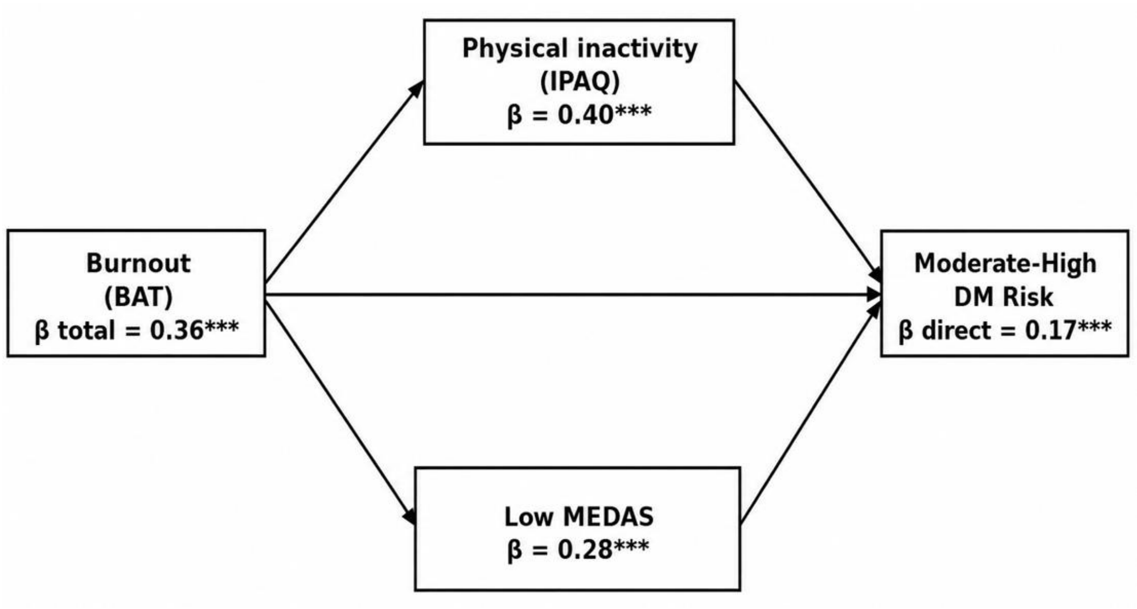

Supplementary Figure S2. Parallel mediation model of the association between burnout and moderate-to-high type 2 diabetes risk according to CANRISK. Physical inactivity and low adherence to the Mediterranean diet were examined as parallel behavioral mediators of the association between burnout and diabetes risk, with adjustment for age, sex, social class, and smoking status. The figure displays the direct effect of burnout on diabetes risk and the indirect pathways operating through each lifestyle behavior. The persistence of a statistically significant direct association after inclusion of both lifestyle variables is compatible with partial statistical mediation. However, because burnout, lifestyle behaviors, and estimated diabetes risk were assessed cross-sectionally, these indirect associations do not establish temporal or causal mediation.
